# Genome-wide analysis of gene expression reveals gene regulatory networks that regulate chasmogamous and cleistogamous flowering in *Pseudostellaria heterophylla* (Caryophyllaceae)

**DOI:** 10.1186/s12864-016-2732-0

**Published:** 2016-05-20

**Authors:** Yan Luo, Jin-Yong Hu, Lu Li, Yin-Ling Luo, Peng-Fei Wang, Bao-Hua Song

**Affiliations:** Key Laboratory of Tropical Forest Ecology, Xishuangbanna Tropical Botanical Garden, Chinese Academy of Sciences, Menglun, Mengla, Yunnan 666303 China; Key Laboratory of Biodiversity and Biogeography, Kunming Institute of Botany, Chinese Academy of Sciences, Lanheiroad 132, Kunming, Yunnan 650201 China; Yunnan Academy of Biodiversity, Southwest Forestry University, Bailongsi 300, Kunming, Yunnan 650224 China; College of biology and chemistry, Puer University, Puer, Yunnan 665000 China; Department of Biological Sciences, University of North Carolina at Charlotte, Charlotte, NC 28223 USA

**Keywords:** RNA-seq, Transcriptome, Cleistogamy, Cleistogamous flowers, Chasmogamous flowers, Environmental response

## Abstract

**Background:**

*Pseudostellaria heterophylla* produces both closed (cleistogamous, CL) and open (chasmogamous, CH) flowers on the same individual but in different seasons. The production of CH and CL flowers might be in response to environmental changes. To better understand the molecular mechanisms of CH and CL flowering, we compared the transcriptome of the two types of flowers to examine differential gene expression patterns, and to identify gene regulatory networks that control CH and CL flowering.

**Results:**

Using RNA sequencing, we identified homologues of 428 *Arabidopsis* genes involved in regulating flowering processes and estimated the differential gene expression patterns between CH and CL flowers. Some of these genes involved in gene regulatory networks of flowering processes showed significantly differential expression patterns between CH and CL flowers. In addition, we identified another 396 differentially expressed transcripts between CH and CL flowers. Some are involved in environmental stress responses and flavonoid biosynthesis.

**Conclusions:**

We propose how the differential expression of key members of three gene regulatory modules may explain CH and CL flowering. Future research is needed to investigate how the environment impinges on these flowering pathways to regulate CH and CL flowering in *P. heterophylla*.

**Electronic supplementary material:**

The online version of this article (doi:10.1186/s12864-016-2732-0) contains supplementary material, which is available to authorized users.

## Background

Cleistogamy is a special mating system of flowering plants and has been reported for approximately 700 species [1–3]. Three categories of cleistogamy are recognized: dimorphic cleistogamy, complete cleistogamy and induced cleistogamy [3]. Dimorphic cleistogamous plants produce both closed (cleistogamous, CL) flowers and open (chasmogamous, CH) flowers on the same individual or on different individuals. The CL flower is often more juvenile in appearance and is characterized mainly by reductions in flower size, corolla and stamen number. A CL flower is also considered to be a modified form of a CH flower [1, 4–7]. Complete cleistogamous plants produce only CL flowers, whereas induced cleistogamous plants can produce both CL and CH flowers but without any floral organ reduction in the CL flowers. The CL flowers are induced due to the failure of the flowers to open when changes in certain environmental factors [3]. In several cleistogamous species, the ability to produce CH and CL flowers can be affected by seasonal and environmental stresses, including drought, flood, salinity, nutrient deprivation, and shade [1, 3, 8–10]. Cleistogamy may be advantageous because CL flowers ensure seed set by self-pollination under severe environmental conditions [1, 11]. The energetic costs (involved in producing sepals, petals and nectar) of CL flowers appear to be considerably lower than those of CH flowers [12, 13].

The genetic regulation and mechanisms of cleistogamy have been studied in several crops that are not naturally cleistogamous. Cleistogamous flowers in barley and rice lack lodicules or display lodicule deformities due to mutations in certain floral identity genes. A mutation in the *ERECT PANICLE2* gene is responsible for the rice cleistogamy mutant *cl7(t)*, in which the lodicules exhibit a weak swelling ability, resulting in the failure of flowers to open [14]. The barley *Cleistogamy1* (*Cly1*) gene is a homolog of A-class *APETALA2* (*AP2*) and is targeted by microRNA miR172. The down-regulation of barley *Cly1* caused by a mutation in the miR172 targeting site represses the development of lodicules, thereby leading to cleistogamy [15]. The *Bn-CLG1A* (*CLG* for cleistogamy) gene encoding a RINGv E3 ubiquitin ligase was isolated from a *Brassica napus*mutant; this gene leads to the formation of petal-closed flowers [16].

However, few studies investigated the genetic regulation of CH and CL flowering patterning in naturally cleistogamous species. The genetic regulatory mechanisms of natural cleistogamy may be quite different from those in crops using rare mutants. In naturally cleistogamous plants, both CH and CL flowers are produced by the same individual exposed to different environments [1–3, 17, 18]. Although the appearance of morphologically reduced CL flowers is not caused by the mutation of certain genes, it may be caused by variations in gene expression in some gene regulatory networks (GRNs) controlling the flowering processes. In a cross-species microarray analysis, Morinage et al. [18] identified 69 genes, including those related to floral development and cold stress response that were differentially expressed in CH and CL flowers of an annual cleistogamous herb, *Cardamine kokaiensis*Yahara.

*Pseudostellaria heterophylla* (Miq.) Pax (Caryophyllaceae) is a native perennial herb widely distributed in north-eastern and eastern China [19, 20], and its roots are used as one of the most popular traditional Chinese medicines. *P. heterophylla* demonstrates typical dimorphic cleistogamy, usually producing both CH and CL flowers on the same individual plant that appear in different positions and indifferent seasons (CH in spring and CL in summer). This species provides an excellent model for investigating the gene regulatory networks that control CH and CL flowering.

In this study, the RNA-Seq platform based on Illumina Hiseq technology was used to compare the transcriptome of *P. heterophylla* CH and CL flowers to investigate differences in gene transcription patterns relating to CH and CL flowering processes. The aims of this study were (1) to identify differentially expressed genes involved in the transition from CH to CL flowering in this dimorphic cleistogamous species, and (2) to investigate putative gene regulatory pathways that might determine differences between CH and CL flowering patterning and processes. Because cleistogamous plants produce CL flowers adapted to severe environmental conditions, our study may help to shed light on the molecular basis for the evolution of environmentally-dependent plant mating systems.

## Results

### Morphological characters of CH and CL flower in *P. heterophylla*

**Fig. 1:**
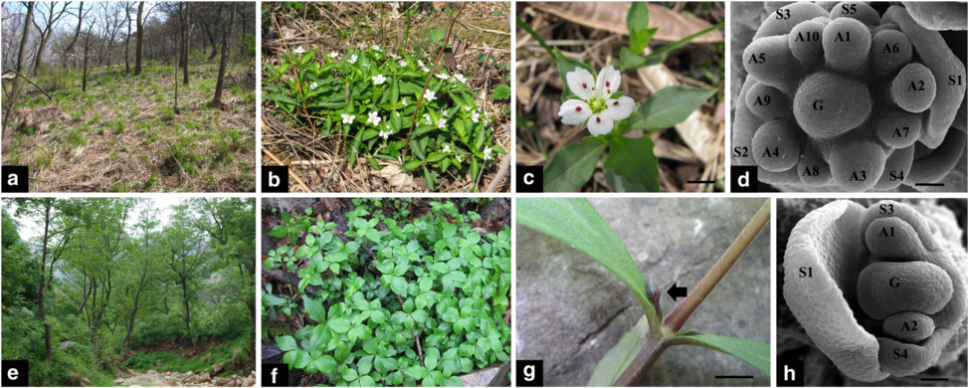
Two types of flower of *Pseudostellaria heterophylla*. **a-d** Chasmogamous (CH) flowering. **a** Habitat of *P. heterophylla* when producing CH flower. **b** *P. heterophylla* individuals with CH flowers. **c** CH flower is at the apical shoot. **d** SEM images of the early stage of CH flower development, with initiation of floral organs (S = sepal, A = stamen, G = carpel, 1–10 indicate the identified number of floral organs). **e–h** Cleistogamous (CL) flowering. **e** Habitat of *P. heterophylla* when producing CL flower. **f** *P. heterophylla* individuals with CL flowers (CL flowers are under the leaves and cannot be seen). **g** CL flower is in a lateral position on the stem (black arrows). **h** SEM images of the early stage of CL flower development, with initiation of floral organs. Bars show 5 mm in **c** and **g**, Bars show 40 μm in **d** and **h**

CH flowers of *P. heterophylla* appear in early spring (late March to late April), when the deciduous trees and shrubs begin to grow new leaves (Fig. 1a). Mature CH flowers develop at the apical shoot and are composed of four whorls of floral organs: five green sepals, five white petals, 10 stamens, and a single ovary with three stigmas (Fig. 1b, c). CL flowers of *P. heterophylla* appear in late spring to summer (late April to late June), after the withering of CH flowers and once light levels fall as the forest canopy forms (Fig. 1e). Mature CL flowers develop at the axils of the first several nodes of the shoot, are smaller than CH flowers and are composed of three whorls of floral organs (usually without a petal whorl) that include four purple-red sepals, two stamens, and a single ovary with two stigmas (Fig. 1f, g). The initiation of floral organ primordia is also different between CH and CL flowers. In CH flowers, 5 sepal primordia, 10 stamen primordia, 5 petal primordiaand 3 carpel primordia are initiated (Fig. 1d), whereas 4 sepal primordia, 2 stamen primordia and 2 carpel primordia are initiated in CL flowers (Fig. 1h) [7].

### Sequencing and assembly

High-throughput sequencing of normalized cDNA libraries resulted in 4,459 million clean pair-end reads for *P. heterophylla* CH flowers and 4,550 million for CL flowers. The raw sequence files have been uploaded to the National Center for Biotechnology Information Sequence Read Archive (http://www.ncbi.nlm.nih.gov/sra/) under the study accession number SRP052597.

Using high-throughput RNA-seq analysis, we identified 44,603 unigenes in the total transcriptome of *P. heterophylla*, with an average length of 860 bp (Table 1). The length distributions of the contigs, transcripts and unigenes are provided in Additional file 1. After strict quality control, the total clean pair-end reads of the two libraries were utilized to reveal transcriptomic information for *P. heterophylla*. Using Trinity, the assembly of clean reads resulted in 218,061 contigs, with an average length of 354 bp, and 27,783 transcripts, with average length of 610 bp. The species that provided most of the top BLAST hits was *Vitis vinifera*, with 9,496 hits, followed by *Populus trichocarpa* and *Ricinus communis*, with 4,724 and 4,269 hits, respectively.

**Table 1:** Characteristics of total assembly data

	Congtigs	transcripts	unigenes
Total Length(bp)	77,330,074	86,706,489	38,415,838
Number	218,061	142,135	44,603
Max Length(bp)	41,978	27,783	27,783
Ave Length(bp)	354	610	860
N50	651	793	1145
>N50 Reads No.	24,500	30,452	9,861
GC %	42.92 %	42.66 %	44.07 %

### Functional annotation

**Fig. 2:**
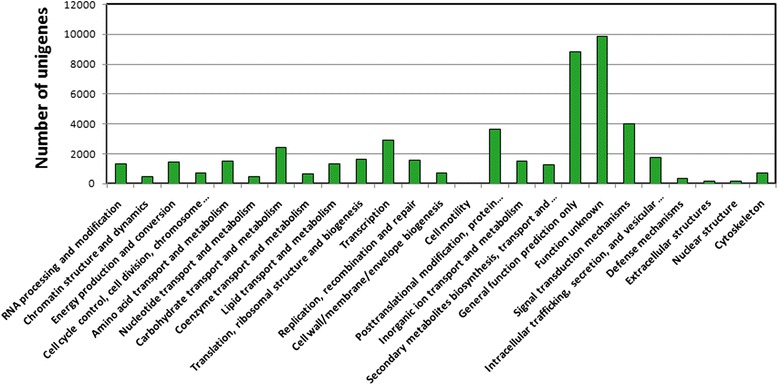
Evolutionary genealogy of genes: Non-supervised Orthologous Groups (eggNOG) analysis. A total of 44,603unigenes were analyzed using eggnog database and subdivided into 25 clusters of orthologous (COG) classifications. x-axis, COG classifications; y-axis, number of unigenes

To obtain functional annotations, all of unigenes were BLASTed against the NCBI non-redundant database, and 32,488 unigenes were searched using BLAST in the Swiss-Prot database (Additional file 2). The functional annotations of *P. heterophylla* genes by the GO, eggNOG and KEGG databases are also shown in Additional file 2. Using the eggNOG database, total unigenes were annotated and subdivided into 25 clusters of orthologous groups (Fig. 2). Among them, 19.93 % (9,854) and 17.90 % (8,850), respectively, of the unigenes obtained from *P. heterophylla* were assigned to “Function unknown” and “General function prediction only”. These categories were followed by a cluster of “signal transduction mechanisms” (4,014; 8.01 %), “Posttranslational modification, protein turnover, chaperones” (3,621; 7.32 %), and “Transcription” (2,922; 5.91 %); the cluster of “cell mobility” (36; 0.07 %) was the smallest group.

### Floral genes are identified

Because one of our aims was to identify candidate genes that are responsible for flowering processes, we specifically searched for *P. heterophylla* homologues of genes and gene families that are known to be involved in flowering time, floral identity, and flower development in *Arabidopsis thaliana*. Candidate homologues were identified as BLAST hits. For the *Arabidopsis* flowering related genes with more than one blast hit to our transcriptome, we chose the longest transcript with the highest identity with *Arabidopsis*. We identified homologues of 428 *Arabidopsis* genes involved in regulating flowering processes, sharing 64–83 % nucleic acid identity with the *Arabidopsis* genes. These candidate flowering-related genes included those affecting flowering time regulation, floral meristem identity, floral organ identity, flower organ development, and establishment of organ and whorl boundaries. Among them were putative homologous genes controlling floral organ identity (ABCE model genes) and some other MADS-box genes (*AGAMOUS-like*), genes controlling flowering time (*FLAVIN-BINDING, KELCH REPEAT, F BOX 1* (*FKF1*), *SQUAMOSA PROMOTER BINDING PROTEIN-LIKE* (*SPL*)), genes involved in meristem size determination and maintenance (*CLAVATA2*, *WUSCHEL* and *SHOOT MERISTEMLESS*), genes involved in organ boundary establishment (*CUP-SHAPED COTYLEDON 1*), and genes involved in controlling organ number, shape, size, and proportions (*PINOID*, *PETAL LOSS*, *ROXY1*)(Additional file 3, the gene names herein are those used in *Arabidopsis*) [21–28].

### Validation of expression of floral genes involved in flowering gene regulatory networks

**Fig. 3:**
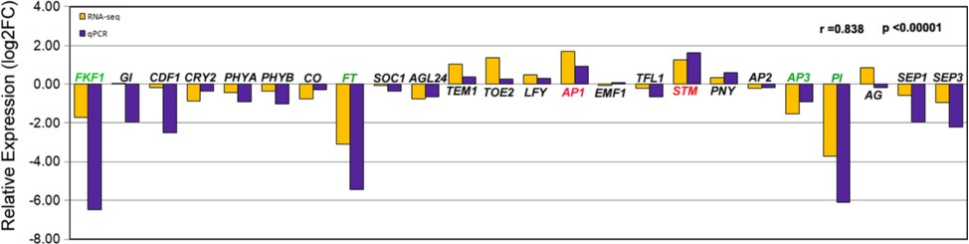
Comparisons of RNA sequencing and qPCR expression data for twenty-four genes associated with flowering process between CH and CL flowers. The Log fold-change (y-axis) in RNA sequencing was estimated based on the data obtained by Reads per kilobase of exon per million mapped reads values (yellow bars). The log fold-change in qPCR were calculated using the comparative CT method (blue bars). We calculated the Pearson correlation coefficient (r)between the different methods for all transcripts. The correlation coefficient was 0.838, *p* <0.00001

The expression of the 24 key candidate genes (Additional file 4) involved in three flowering regulatory modules was validated using qRT-PCR (Fig. 3). The expression patterns of most of loci were consistent with transcriptome sequencing based on the Pearson correlation coefficient value (*r* = 0.838). *FKF1*, *FT*, *STM*, *AP1*, *AP3*, and *PI*, which were expressed differentially between CH and CL flowers, were detected both by RNA-seq and qRT-PCR (Fig. 3). The remaining flowering genes such as *CRY2, PHY, CO, SOC1, AGL24*, etc. were not expressed differentially between CH and CL flowers. The expression of *GI* and *CDF* were not considered differentially expressed between CH and CL flowers as they were not consistent between RNA-sequencing and qRT-PCR. The expression variation of these genes may play important roles in determining CH and CL flowering, however, as we discuss below.

### Differentially expressed transcripts abundance

Comparing the RNA-seq data between CH and CL flowers, we found a total of 396 genes were differentially expressed using the strict cutoff thresholds (fold-change >4, FDR <0.05); Of this total, 93 were up-regulated in CH flowers, 303 were up-regulated in CL flowers, three transcripts were expressed only in CH flowers, and 57 were expressed only in CL flowers (Additional file 5).

**Fig. 4:**
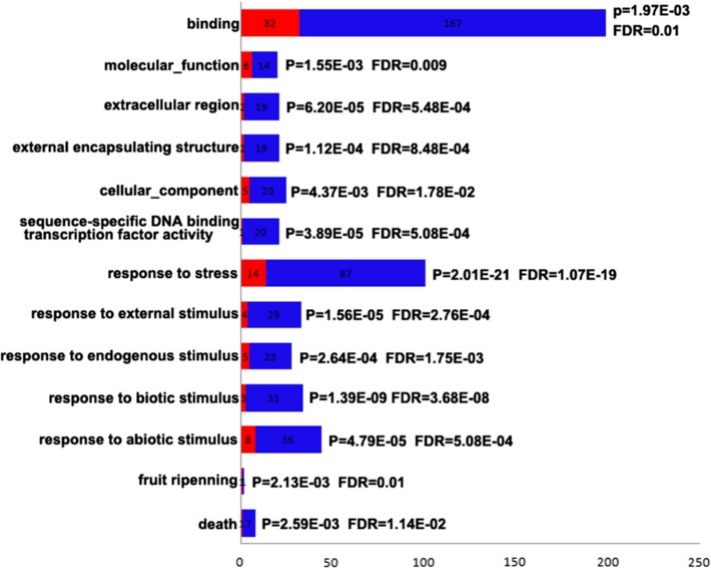
Distributions of differentially expressed transcripts (DETs) (x-axis) into Gene Ontology (GO) categories (y-axis) according to GO enrichment analysis. Up-regulated transcript numbers were shown in bars, red bars representing up-regulated in CH flowers and blue bars representing up-regulated in CL flowers. A *p*-value and a false discovery rate (FDR) were given for significance

The GO enrichment analyses showed that the differentially expressed transcripts (DETs) were enriched in biological process, cellular components and molecular functions (Fig. 4 and Additional file 6) using cutoffs of FDR <0.05. A total of thirteen enriched GO terms were identified, including eight of biological process, three of cellular components and two of molecular functions (Fig. 4). Further, the biological process are related to the response to biotic and abiotic stimulus, the response to endogenous stimulus, the response to external stimulus, the response to stress, and sequence-specific DNA binding transcription activity were attributing to the significant enrichment (p-value <10^−3^). Moreover, cellular component associated with external encapsulating structure and extracellular region, and molecular function associated with molecular function and binding, were attributing to the enrichment (Fig. 4).

To identify significantly enriched KEGG categories for the DETs from the CH and CL libraries, we assigned 171 DETs to the KEGG database. The details of the KEGG classification are presented in Additional file 7 and 8. The major pathways identified were “metabolism”, “biosynthesis of secondary metabolites”, “hormone signal transduction”, and “plant-pathogen interaction”.

## Discussion

**Fig. 5:**
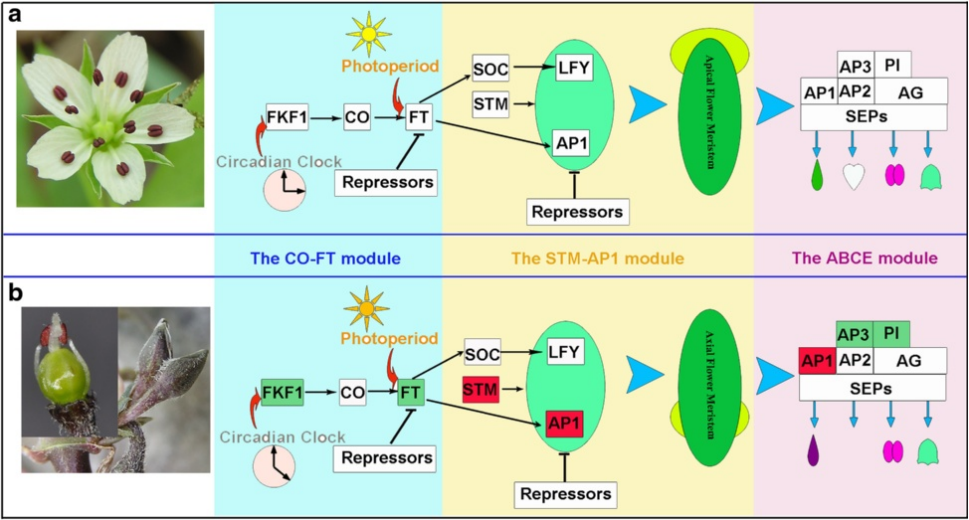
The putative gene regulatory networks (GRNs) that regulate the production of chagamous (CH) and cleistogamous (CL) flowering in *Pseudostellaria heterophylla*. Genes represented by blocks. Lines with an arrow represent promotion, and those with a perpendicular bar represent repression. **a** GRNs in CH flower production. **b** GRNs in CL flower production. Gene expression variation represented by different colour in CL flowering GRNs: gene expression level not to change is shown in white; gene expression level up-regulated is shown in red; gene expression level down-regulated is shown in green. *FKF1*down-regulated due to the change of Circadian Clock, *FT* down-regulated due to the poor light quality, the floral identity gene *AP1*up-regulated due to *STM* promotion. *AP3* and *PI* down-regulated due to down-regulated upstream genes

This is the first work to analyse the flowering GRNs with RNA-seq based transcriptomes in a naturally cleistogamous plant to understand the genetic mechanisms for different flower patterning. Gene expression controlling development can be modified as a consequence of environmental changes, leading to altered multiple flower developmental pathways. Three modules are proposed to determine CH and CL flower process in natural populations (Fig. 5).

### The CO-FT module regulates CH and CL flowering time

Day length and the quality of light play a significant role in the floral transition in many plants [21, 22, 25, 28]. The CO-FT module was proposed to explain how plants might initiate flowering in response to photoperiod [21, 22, 28, 29], and this module is highly conserved across plant species [22]. In *Arabidopsis*, the expression of *FT* is regulated by the CO protein and is a crucial aspect of photoperiodic flowering. *CO* expression is associated with the combined action of photoreceptors (*PHYA*, *PHYB*, and *CRY2*) and the circadian clock system (*FKF1* and *GI*) [30–33]. Further, the protein FT, which acts as a universal florigen [34–38], is a major target of CO. Transport of the transcription factor FT from the leaves to the shoot apical meristem results in the activation of transcriptional cascades that specify the transition from vegetative to reproductive growth.

Homologues of most genes associated with the CO-FT module were identified in the *P. heterophylla* transcriptome, including *FKF1*, *GI*, *CO*, *FT*, and other factors. Interestingly, the upstream gene*FKF1* and the downstream gene *FT* of the CO-FT module both showed lower expression levels in CL flowers than in CH flowers (Fig. 5). *FKF1* has been reported to play a key role in regulating the circadian clock and photoperiodic flowering [30–33]. Searle et al. [39] reported that reducing expression of *FT* indirectly repressed expression of genes involved in floral induction at the meristem and delayed flowering time in *Arabidopsis*. The lower levels of *FKF1* and *FT* expression might play important roles in the delay of flowering time in CL flowers in our species. Natural *P. heterophylla* populations usually grow under temperate forests. CH flowers are produced only in early spring when the deciduous trees and shrubs begin to grow new leaves; the appearance of CL flowers appears a few weeks later, and light levels fall as the forest canopy forms. The photoperiod and circadian clock changes from those in spring might regulate *FKF1* expression and further regulate *FT* gene expression in CL flowers.

### The STM-AP1 module regulates CL flower meristem specification

The specification of FM is crucial for successful flower development [40–43]. Two FM identity genes, *AP1* and *LFY*, are conserved in their FM specification function [42, 44–46], and are regulated by multiple factors (Fig. 5).

Homologues of these FM identity genes and their regulatory factors were also identified in the *P. heterophylla* transcriptome. Surprisingly, although *FT* and *SOC1* expression was reduced in CL flowers, their targets*AP1* and *LFY* were up-regulated. *AP1* is highly expressed in CL flowers, which makes it a good candidate for contributing to the spatial specification of FM initiation. We found that *STM*, which directly activate*AP1* and *LFY*, was significantly up-regulated in CL flowers (Fig. 5). In *Arabidopsis*, *STM* acts to regulate flower patterning, branching, and internode growth and the specification of axillary meristems during inflorescence development [47–51]. In *P. heterophylla*, CL inflorescences develop in the axils of the first several nodes of the shoot. Unlike CH flower production at the apical meristem, the axillary flower meristems may play a crucial role in the establishment of a CL inflorescence, ultimately leading to the formation of CL flowers. We speculate that *STM* plays a fundamental role in promoting the formation of CL FMs in the axils of cauline leaves in *P. heterophylla*.

### The ABCE model regulates CH and CL floral patterning

The classic ABC (or ABCE) model proposed to determine floral organ identity is conserved across angiosperms [25–27]. Four classes of genes (A, B, C, and E) are well known to act in combination for proper organ formation in each whorl [52]. All of the floral organ identity genes homologous to those of *Arabidopsis* A, B, C, and E genes were also identified in *P. heterophylla* (Fig. 5). The CH flower of *P. heterophylla* is a typical eudicot pentamerous flower with four whorls: five sepals, five petals, ten stamens and three carpels. However, the *P. heterophylla* CL flower displays floral organ reductions: sepals are reduced to four; petals are absent; stamens are reduced to two; and carpels are reduced to two (Fig. 1d, h) [7]. The A class gene *AP1* is highly expressed in CL flowers. *AP1* has two functions, floral meristem specification and perianth identity, and these two functions may not be separable [41, 53, 54]. Our previous study found the significant difference in the initiation sequence of sepals in CH vs CL flowers of *P. heterophylla*: five sepal primordia are initiated one by one in a 2/5 helix sequence in the CH flower; in contrast, four sepal primordia in the CL flower initiate alternatively in pairs [7]. The four sepals of the CL flower are not derived from sepal reduction of the pentamerous CH flower but may arise as a continuation of bracteole initiation [7]. Increased levels of *AP1* expression may strengthen the floral meristem specification function of *AP1*, resulting in bracteole-like sepal formation in the CL flower.

The B class genes *AP3* and *PI* are significantly decreased in CL flowers. *AP3* and *PI* are closely related MADS paralogous lineages, and their functions in establishing the identity of petals and stamens in floral development are conserved in both eudicots and monocots [55, 56]. In our previous floral organogenesis study [7], we found that, in floral organ identity stages, fewer primordia of stamen and absence of petal primordia in CL flowers (Fig. 1d, h), which might explain the stamen number reduction and absence of petals compared to CH flowers. Mondragon-Palomino et al. [57] found that the complex perianth in orchids is patterned by differential expression of multiple B-class gene paralogs. Zhang et al. [58] suggested that disruption of the petal identity gene *AP3-3* is highly correlated with loss of petals in the buttercup family (Ranunculaceae). Thus, the reduced stamen number and absence of petals in CL flowers in our species are likely related to the decreased levels of expression in B class genes (Fig. 5b). However, further investigations for ABCE functions in CL and CH flower patterning are needed.

### Other differentially expressed genes in CH and CL flowering

Except for those flowering related genes, 369 transcripts were considered significantly differentially expressed between CH and CL flowers. These differentially expressed genes include those related to environmental stress responses, plant defence response, plant hormone signal transduction, plant development and growth and secondary metabolite biosynthesis. Especially, genes involved in stress response were significantly overrepresented (*p*-value = 2.01E-21, Fig. 4), with14 and 87 stress-response genes up-regulated in CH and CL flowers, respectively. We could not rule out the possibility that the expression differences of stress-related genes could be a result of the specific experimental design. Nevertheless, sixteen transcripts with homology to the *HSP* family such as *HSP70*, *HSP83*, *HSP90*, and some *small Heat Shock Protein* (*sHSP*) families were detected in CL flowers (Additional file 5, marked with red). *HSP* induction may reflect differential physiological responses to environmental stresses, such as drought, cold, and high temperature [59–61]. In a previous study, two *HSP* genes were reported to be up-regulated with cold treatment in *Cardamine kokaiensis* CL flowers [18]. Further, eight homologues of *WRKY* transcription factor genes (Additional file 5, marked with purple), which are important regulators of active biotic and abiotic stress responses [62], were significantly up-regulated in CL flowers. It is also evident that some members of the *WRKY* family may play important regulatory roles in promoting flowering [63–65].

With the KEGG analysis, we found nine genes related to flavonoid biosynthesis pathway were significantly up-regulated in CL flowers (Additional file 5, marked with orange). These included encoding enzymes involved in flavonoid and anthocyanin syntheses, such as *Anthocyanidin Synthase*, *Flavonoid 3′-hydroxylase*. Flavonoids are important secondary compounds and the main pigments in flowers [66, 67]. The blue and purple flower colours are derived from flavonoids and anthocyanins [68–70]. The up-regulation of genes related to flavonoid and anthocyanin biosyntheses in CL flowers may result in the purple colour of the sepals, instead of the white colour of the petals in CH flowers.

## Conclusions

This is the first work to investigate the flowering GRNs in a naturally cleistogamous plant. Gene expression patterns are modified as a consequence of environmental changes, leading to altered multiple flower developmental pathways. We proposed three modules that regulate flowering time, meristem development and patterning in a natural populations of *P. heterophylla*. Even though investigations of the spatiotemporal expression patterns of these genes are required to assess their functions in determining CH and CL flowering, our study provided a foundation for dissecting the molecular basis of flowering time and patterns in naturally cleistogamous plants. Cleistogamous plants can be a very important model for studying the adaption and evolution of flowering behaviour.

## Methods

### Plant materials

Materials for the transcriptome sequencing study were collected from a natural population from Mount Lao, located near the East China Sea on the south-eastern coastline of the Shandong Peninsula in China (120°36′10″E, 36°06′27″N). CH flower buds were collected on 8 April, 2014 (average minimum temperature is 8 °C, average maximum temperature is 15 °C and average precipitation is 36 mm in April; http://qingdao.tianqi.com/laoshan/). CL flower buds were collected on 26 May, 2014 (average minimum temperature is 13 °C, average maximum temperature is 20 °C and average precipitation is 54 mm in May; http://qingdao.tianqi.com/laoshan/). Both CH and CL whole dichotomous inflorescences were collected around 9:00 am −10:00 am, with each inflorescence including 3–5 flower buds at different developmental stages. The first flower bud of a dichotomous inflorescence (the oldest one) is well-developed with all of floral organs about 5 mm in size in the CH flower bud and 3 mm in the CL flower bud. The second flower bud is younger than the first and the third flower bud is the youngest of all. In most cases, there are 1–2 barely-visible flower buds located at the bottom of bract. CH and CL flower buds were collected fresh, frozen immediately in liquid nitrogen and then were transported in dry ice to a freezer where they were stored at −80 °C.

### Sequencing

CH and CL flower libraries were generated from flower buds, and each flower bud sample consisted of a mixture of flower buds from different developmental stages obtained from 50 individuals of the same *P. heterophylla* population. For each kind of flower, total RNA was isolated from the flower buds using Trizol Reagent (Invitrogen) and treated with DNaseI (Promega) to remove residual contaminating DNA. The quality and integrity of the RNA were determined using an Agilent 2100 Bioanalyzer (Agilent). Two RNA-seq libraries (e.g. CH flowers and CL flowers) were prepared using the Illumina TruSeqRNA Sample Preparation Kit (Illumina) following the manufacturer’s instructions. Two normalized cDNA libraries (CH flowers, CL flowers) were constructed and sequenced using the Illumina HiSeq platform (Shanghai Personal Biotechnology Co. Ltd, China) to generate 100 bp paired-end raw reads.

### Assembly and functional annotation

Clean reads were obtained by removing adaptor sequences, low-quality reads, and shorter reads (shorter than 50 bp). Trinity (http://trinityrnaseq.github.io) was used to assemble the quality reads into contigs and transcripts [71]. Basic Local Alignment Search Tool (BLAST) was utilized for searching sequence alignments (E-value <1.00E-5) between unique sequences and the non-redundant NCBI (http://www.ncbi.nlm.nih.gov/) and Swiss-Prot (http://www.expasy.ch/sprot) databases. The best-hit transcripts were selected as unigenes. The unigenes with functional categories were annotated by the Gene Ontology database (GO, http://www.geneontology.org) [72], Non-supervised Orthologous Groups (eggNOG) database (http://eggnog.embl.de/) [73], and the Kyoto Encyclopedia of Genes and Genomes database (KEGG, (http://www.genome.jp/kegg/) [74].

### Identification of differentially expressed transcripts

Reads per kilobase of exon per million mapped reads (RPKM) [75] were used to normalize the abundance of transcripts on the basis of eliminating the influence of different gene lengths and sequencing discrepancies. Transcripts differentially expressed between the CH and CL flower libraries were identified using DEseq [76]. DETs between two libraries were required to have an absolute value of a log_2_foldchange (log_2_FC) > 2 in expression, a *p*-value of <0.001and an FDR <0.05 [77, 78].

The DETs were subjected to GO and KEGG Orthology (KO) enrichment analysis on the basis of a hypergeometric test (cutoff: FDR <0.05). The KEGG database was also used to predict the pathways in which the DETs were involved, and the pathway annotation was identified by the KEGG mapper (http://www.genome.jp/kegg/tool/map_pathway2.html).

### Identification of genes involved in flowering processes

A total of 599*Arabidopsis* genes associated with gene regulatory networks controlling flowering processes were obtained from GO terms (GO: 0009908 flower development, GO: 0009909 regulation of flower development, GO: 0048439 flower morphogenesis, GO: 0048438 flower whorl development; GO: 0048440 carpel development, GO: 0010093 specification of floral organ identity, GO: 0048497 maintenance of floral organ identity, etc., http://amigo.geneontology.org/) as well as from Srikanth & Schmid [22] and Alvarez-Buylla et al. [24]. The sequences of these genes were downloaded from the Arabidopsis Information Resource (TAIR, http://Arabidopsis.org/). We used the *Arabidopsis* genes involved in flowering processes as query sequences to search potential homologous genes among the transcript sequences of *P. heterophylla* by stand-alone BLAST (2.2.30+ version, ftp://ftp.ncbi.nlm.nih.gov/blast/executables/blast+/LATEST/) with an E-value <1.00E-10. All hits with identities above 60 % were considered to be homologous genes in *P. heterophylla*.

### Validation of floral genes by quantitative real-time PCR

Twenty-four transcripts related to gene regulatory networks of flowering processes identified in the transcriptome sequencing analysis were validated and quantified using quantitative real-time PCR (qRT-PCR). Primers were designed using Primer Premier 5 according to transcriptome sequencing data (Additional file 4). Total RNA was obtained from the biological replicates of those samples used for RNA sequencing. Reverse cDNA was synthesized using PrimeScript Reagent Kit with gDNA Eraser (Takara), and RT-PCR was performed using an Eppendorf Master cycler. *P. heterophylla β-actin* was used as an internal control to normalize the expression level, and all experiments were performed with three repeats. The reaction was carried out in a total volume of 10 μL containing 5 μL SYBR Green Master Mix (Takara), 1 μL diluted cDNA mix, 0.5 μL each primer (10 mM), and 3 μL RNase-free water. The reactions were performed in a LightCycler480 real-time PCR system (Roche) at 95 °C for 30 s, followed by 45 cycles of 95 °C for 5 s and 60 °C for 30 s. Amplification and detection of only one PCR product was confirmed by performing melting curve analysis of the amplification products at the end of each PCR. After the PCR program, the expression level of different genes was analysed using the comparative CT method (2^-△△CT^ method) [79].

### Availability of supporting data

The sequence datasets supporting the results of this article are available from the Short Read Archive (SRA) database at NCBI under the accession SRP052597.

## Additional files

Additional file 1: Distribution of contig lengths (a), transcripts lengths (b) and unigene lengths (c). (PDF 556 kb) Additional file 2: Transcripts annotated by GO, KEGG, and eggNOG. (XLSX 4808 kb) Additional file 3: Putative homologues of Pseudostellaria heterophylla genes involved in flowering process identified by BLAST search with Arabidopsis genes. (XLSX 56 kb) Additional file 4: 24 floral genes of quantitative real time PCR for validation of RNA-Seq data. (XLSX 13 kb) Additional file 5: Differentially expressed transcripts between CH and CL flowers. In total, 396 genes were identified as differentially expressed (|log2FC| > 2, *p*-value < 0.001 and FDR < 0.05) between CH and CL, including 93 genes that were up-regulated in CH (pink shadow), and 303 genes that were up-regulated (green shadow) in CL. (XLSX 184 kb) Additional file 6: GO Enrichment analysis of differentially expressed transcripts. (XLSX 26 kb) Additional file 7: KEGG Enrichment analysis of differentially expressed transcripts. (XLSX 12 kb) Additional file 8: KEGG pathways classification for differentially expressed transcripts between CH (A) and CL (B) flowers. Red dot represented transcripts up-regulated in CH flowers, green dot represented transcript up-regulated in CL flowers. (XLSX 36 kb)

## Abbreviations

CL: cleistogamous
CH: chasmogamous
DETs: Differentially expressed transcripts
qRT-PCR: quantitative real-time PCR
BLAST: Basic Local Alignment Search Tool
GRNs: gene regulatory networks
FM: flower meristem
