# Supplementary figures and images for: Genome-wide analysis of gene expression reveals gene regulatory networks that regulate chasmogamous and cleistogamous flowering in Pseudostellaria heterophylla (Caryophyllaceae)
Source: BMC Genomics. 2016 May 20;17:382. doi: 10.1186/s12864-016-2732-0 (PMC4875749; doi:10.1186/s12864-016-2732-0)

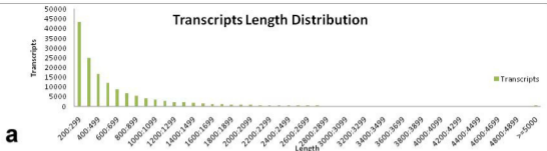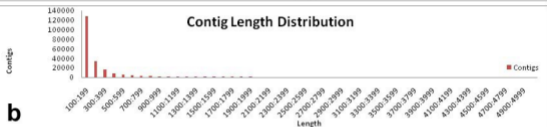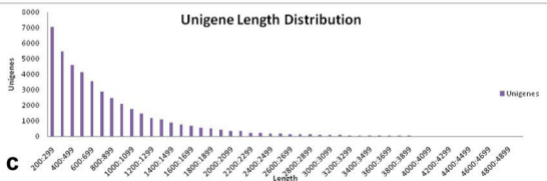

**Additional file 1 Distribution of contig lengths (a), transcripts lengths (b) and unigene lengths (c).**

Supplement: Additional file 1: — Distribution of contig lengths (a), transcripts lengths (b) and unigene lengths (c). (PDF 556 kb) [file 12864_2016_2732_MOESM1_ESM.pdf]
